# The Statistical Report of King Edward's Hospital Fund. IV

**Published:** 1914-01-31

**Authors:** 


### HOSPITAL ECONOMICS.

(Inquiries and Correspondence are invited.)

### The Statistical Report of King Edward's Hospital Fund.*—IV.

It was intended in this series of papers to deal with the importance of accuracy and uniformity in allocating certain expenditure to in-patients and to out-patients. In a recent issue (The Hospital, December 20) this matter was touched upon when the Supplementary Memorandum issued by the Funds was reviewed, and a plea made for assessment of a rate per out-patient attendance. But the adoption of such an arrangement is a matter for future decision; for the present, taking it that with the further assistance given by the Memorandum the division will be made as fairly as circumstances allow, we can only strive for accuracy and uniformity in sub-division. Here the accountant will derive additional benefit from the excellent work of the committee of secretaries in the amplification of the index of classification contained in the Memorandum. However, the allocation of many items of expenditure often requires much thought and judgment, and as decisions must be left to individual opinion we must, while examining with interest comparisons of sub-divisions of expenditure, rely on the gross cost of material on which to base conclusions.

The suggestion of assessment has been made in respect of the deduction of the cost of out-patients from the main outgoings; other improvements of the Statistical Report can doubtless be found. There are two matters especially worthy of consideration. One is the inclusion of the total number of all paid employees. This fact will explain a great deal in connection with expenditure under two, at least, of the headings—namely, "Provisions" and "Salaries and Wages." As the size of the staff is often greatly influenced by the convenience of the hospital buildings, this suggestion leads us to another—that some indication of the age of the fabric may be given. In London our hospitals may be nearly all described as either "old," "composite," or "modern," and the first letter of one or other of these words, following the title of the hospital in the tables, would occupy small space and give valuable information. We might ask, too, for the date of foundation, as tradition has often a great bearing on expenditure.

### The Payment of Employees.

The publication of such a document as that under review must lead to a large amount of interchange of useful information between the hospitals, and one of the subjects upon which the circulation of data is particularly desirable is that of the rates of remuneration of the different classes of employees. Of course, one of the most interesting and valuable features of the voluntary system is the fact that it is possible to choose an appropriate staff to fit the needs of a particular institution, and that according to the degree of responsibility there is considerable variation in the value of appointments. This is a good thing; the prospect of promotion gives incentive to an officer to carry out his duties efficiently. But comparison of information will reveal the fact that for what is to all intents and purposes the same work, both in character and amount, there is frequently an appreciable difference between the institutions in the remuneration of certain grades of employment. The Hospital Officers' Association has, we believe, done some useful work in providing information as to rates of pay of groups of employees, but recognising that the task is by no means a light one, it seems reasonable to hope that the machinery set up by King Edward's Fund may be used to produce further comparative figures in respect of this matter; it would not be necessary to publish these annually, but, say, once in every three years.

The Statistical Report contains a useful section giving particulars of the laundry expenses of 106 hospitals. From these we learn that sixteen institutions possess their own laundries, and the cost per occupied bed (excluding the London Fever Hospital) ranges from £3 15s. 8d. to £9 9s. 3d. Nine hospitals do a certain amount of washing on the premises and send the rest out; in this group the cost varies from £3 12s. 1d. to £8 12s. 4d. The remainder, being, of course, the larger proportion, show a range from £1 14s. 2d. to £10 13s. 11d. per occupied bed.

### True Test of a Hospital Laundry.

To compare type with type, Guy's Hospital, with its own laundry, shows a cost of £6 19s. 11d.; and St. Thomas's, sending the washing out, £7 7s. 11d. Other examples could be given; but, generally speaking, from the actual cash result, taking into consideration the special requirements of each institution, there is no startling divergence to be found.

The figures as to laundry cost should be prepared in an impartial manner in the cases where its own washing is done by a hospital. The institution is naturally desirous of showing successful and economical working in each department. We do not say that the figures before us have not been calculated by persons with an entirely detached point of view, but it is difficult to lose sight of the possibility that certain charges, managerial and other, have provided problems which all institutions have not solved in an identical manner.

The common experience of many institutions that the modern methods of treating laundry articles are responsible for a large amount of wear and tear leads one to hope that possession of its own laundry enables the institution to work on lines exclusively suitable to its own requirements.

* Previous articles appeared on November 29 and December 13, 1913, and January 17, 1914.

This being so, it follows that from the point of view of economy there is a financial result which is not revealed in any statement of the cost of washing, but to be found in that part of the accounts referring to expenditure on linen and similar supplies. No final criticism on the advantage or disadvantage of a hospital laundry is possible without full information throwing light on this point.

Section 4 of the Statistical Report consists of a table showing the highest and lowest prices paid for a number of articles in use by 107 hospitals on December 31, 1912. There is a very necessary note at the foot of the table stating that "the circumstances under which goods are purchased, the quantities and qualities, the date and length of contract, and conditions of delivery would have to be taken into consideration before assuming that the lowest prices paid by a single hospital in any year were obtainable by every institution," and in the absence of such data it is difficult to form any opinion on the basis of the particulars given, or to understand why the figures are provided. As instances, it is obvious that the chloroform at 1s. 1¼d. per pound is an entirely different article from that at 8s. 1d.,* and that quality must be an explanation of the wide variation in the prices of clinical thermometers.

* For a full discussion of the point thus alluded to reference should be made to an exhaustive article on this topic in The Hospital (November 16, 1912, page 181).

